# Perceiving where another person is looking: the integration of head and body information in estimating another person’s gaze

**DOI:** 10.3389/fpsyg.2015.00909

**Published:** 2015-06-30

**Authors:** Pieter Moors, Filip Germeys, Iwona Pomianowska, Karl Verfaillie

**Affiliations:** ^1^Laboratory of Experimental Psychology, Katholieke Universiteit Leuven, Leuven, Belgium; ^2^Faculty of Economics and Business, Katholieke Universiteit Leuven, Leuven, Belgium; ^3^The Leon Schiller National Higher School of Film, Television and Theatre, Lodz, Poland

**Keywords:** gaze perception, perceived gaze direction, social attention, joint attention, overshoot effect, head–body orientation, implied motion

## Abstract

The process through which an observer allocates his/her attention based on the attention of another person is known as joint attention. To be able to do this, the observer effectively has to compute where the other person is looking. It has been shown that observers integrate information from the head and the eyes to determine the gaze of another person. Most studies have documented that observers show a bias called the overshoot effect when eyes and head are misaligned. That is, when the head is not oriented straight to the observer, perceived gaze direction is sometimes shifted in the direction opposite to the head turn. The present study addresses whether body information is also used as a cue to compute perceived gaze direction. In Experiment 1, we observed a similar overshoot effect in both behavioral and saccadic responses when manipulating body orientation. In Experiment 2, we explored whether the overshoot effect was due to observers assuming that the eyes are oriented further than the head when head and body orientation are misaligned. We removed horizontal eye information by presenting the stimulus from a side view. Head orientation was now manipulated in a vertical direction and the overshoot effect was replicated. In summary, this study shows that body orientation is indeed used as a cue to determine where another person is looking.

## Introduction

Human primates use a plethora of visual cues to direct their attention. Some of these are social in nature, like another person’s attention. The process through which an observer allocates his/her attention based on the attention of a looker has also been referred to as joint attention (Emery, 2000). This process of joint attention allows humans to gain insight in the mental state (Baron-Cohen, 1994) and intentions of conspecifics (Manera et al., 2010), and also has been shown to influence language processing (Hanna and Brennan, 2007; Staudte and Crocker, 2011; Staudte et al., 2014). Furthermore, an individual looking at the observer can be a cue for social interaction (Argyle and Cook, 1976; Pönkänen and Hietanen, 2012) while an averted gaze could indicate another emotional relation. Lastly, the location at which another person is looking can signal the presence of potentially interesting or threatening objects in the environment.

Essentially, these processes of determining another person’s attention boil down to computing gaze direction of the other person. Indeed, Bayliss et al. (2011) have recently obtained evidence suggesting a direct link between perceived gaze direction and social attention. That is, they showed that adapting to gaze direction attenuated gaze cueing effects.

The importance of gaze perception for the human visual system is reflected in the fact that it involves different neural pathways than face-selective processing. For example, cells in the superior temporal sulcus (STS) have been shown to be responsive to perceived gaze direction in general (Perrett et al., 1992; Carlin et al., 2011), direct gaze has been shown to activate the amygdala (George et al., 2001) and neuropsychological studies have indicated that prosopagnosics show normal gaze discrimination and adaptation (Duchaine et al., 2009).

Several factors have been documented to play an influential role in perceiving gaze direction. For example, manipulating contrast polarity (Ricciardelli et al., 2000; Sinha, 2000) or luminance (Ando, 2002, 2004) of the eye region has detrimental influences on accurately perceiving gaze direction. Moreover, determining another person’s gaze does not solely depend on information available from the eyes. It involves integrating several sources of information available from the face and body. For example, it has been reported that head orientation (Wollaston, 1824; Gibson and Pick, 1963; Cline, 1967; Anstis et al., 1969; Noll, 1976; Maruyama and Endo, 1983; Masame, 1990; Todorović, 2006), nose angle (Langton et al., 2004), face eccentricity (Todorović, 2009), eyebrows (Watt et al., 2007), the epicanthal fold (West et al., 2000), iris color (West, 2011), and monocular or binocular eye information (West, 2010) all modulate perceived gaze direction.

Generally, observers are quite accurate in determining where another person is looking. Indeed, some studies have shown that gaze acuity (the threshold for detecting a difference in gaze direction) is quite high (Gibson and Pick, 1963; Symons et al., 2004; Bock et al., 2008). Some biases in perceived gaze direction have been reported however, especially with respect to integrating eye and head information. That is, when the head is not oriented straight to the observer, perceived gaze direction is sometimes shifted in the direction opposite to the head turn. Because this shift in perceived gaze direction is in the direction opposite to the head orientation, this effect has also been called the overshoot or repulsion effect (Gibson and Pick, 1963; Anstis et al., 1969; Noll, 1976; Masame, 1990; Langton et al., 2004; Todorović, 2006, 2009). An explanation for this overshoot effect was suggested by Anstis et al. (1969) and Otsuka et al. (2014), more recently. That is, when head orientation is varied while the eyes keep fixating at the same point in space, the position of the pupil rotates in the direction opposite to the head. Anstis et al. (1969) argued that this change of the amount of visible sclera to both sides of the pupil is the primary determinant of the overshoot effect.

In sum, an extensive literature exists on estimating gaze direction and the modulatory nature of several variables (especially head orientation) on perceived gaze direction. Nevertheless, it is possible that not only information from the eyes and head act as a cue to judge gaze direction and, accordingly, guide attention (Perrett et al., 1992; Hietanen, 2002; Seyama and Nagayama, 2005). Indeed, the whole body might also play a prominent role. However, this issue has been addressed to a limited extent in the literature.

Direct behavioral evidence for the hypothesis that body information influences perceived gaze direction is sparse. The available evidence mainly comes from indirect behavioral paradigms like cueing and Simon paradigms and neurophy-siological studies. The gaze cueing literature frequently relies on the classical Posner cueing paradigm in which a cue is presented either centrally or peripherally causing participants to respond faster to a target on the location congruent with the cue than incongruent with the cue. Presenting a face with a certain gaze direction has been shown to reflexively direct attention to the gazed-at location (Friesen and Kingstone, 1998; Driver et al., 1999). Hietanen (2002) manipulated head and body orientation while keeping the eyes and head aligned and reported an influence of body orientation on reflexive attention orienting. That is, when head and body were misaligned (i.e., incongruent) a cueing effect was found in contrast with no cueing effect for congruent head and body orientations. Seyama and Nagayama (2005) extended these findings by independently manipulating eye, head, and body orientation and asking participants to decide whether a computer-generated character was looking at the observer or not. Again, an effect of body orientation was shown. Pomianowska et al. (2012) have recently provided additional evidence for the role of body orientation in attentional orienting using a Simon task. The Simon effect refers to the observation that observers are generally faster and more accurate when the stimulus is presented in the same relative location as the response, irrespective of the relevance of the stimulus location to the task. Their findings were in accordance with Hietanen (2002) in that a Simon effect was found only for incongruent head and body orientations. That is, a body oriented to the left of the observer with a head oriented straight at the observer elicited a faster response at the right rather than the left response location and *vice versa* for bodies oriented to the right of the observer. The observation that congruent eye/face or face/body orientations did not result in attentional cueing effects could be due to the fact that an averted head or body with a congruently averted gaze is a less powerful social signal in that the observed person is interpreted to be less related to the observer. That is, according to Hietanen, if multiple cues (eyes, head, and body) are processed in an allocentric frame of reference (i.e., related to the observed person), aligned eyes and head or head and bodies suggest no attentional shift from the observed person whereas misaligned eyes and head or head and body do suggest an attentional shift from the observed person.

These psychophysical findings have been backed up by neurophysiological evidence suggesting that there are cells responsive for body posture in the macaque STS (Wachsmuth et al., 1994; Jellema and Perrett, 2003). Furthermore, these cells respond to specific conjunctions of eye, head, and body posture. Based on these findings, Perrett et al. (1992) proposed a hierarchical model for the neuronal responses in the macaque STS in which information from the eyes can override head information which, in turn, can override body information.

As is apparent from the above-mentioned studies, most of them relied on indirect attentional measures to study the influence of body orientation on perceived gaze direction. The goal of the present study was to provide direct behavioral evidence for the question whether body information is integrated with head information to determine another person’s gaze direction. Specifically, the aim was to examine whether the information from the head and body is integrated in a similar manner as eye and head information for perceiving another person’s gaze. That is, we examine gaze estimation rather than gaze cueing. It is important to note that the focus of the present study is on perceived gaze direction (more specifically, the influence of body orientation on perceived head orientation), not on the perception of *eye* orientation *per se*. In fact, our stimuli would not be suitable to properly study perceived eye orientation since the eye region in our stimulus was very small, making eye orientation information hardly available, and the eyes were always aligned with the head such that any information derived from the eyes would always be congruent with the orientation of the avatar’s head.

## Experiment 1

In Experiment 1, we manipulated head and body orientation in a factorial way. The eyes were always aligned with the head. We hypothesized that an overshoot effect would be observed similar to the effect reported in Anstis et al. (1969) and Gibson and Pick (1963) when the integration of head and eye information was studied. In addition, eye movements were monitored for two reasons. First, previous studies on gaze estimation relied on manual responses of the participant to measure perceived gaze direction. If gaze following indeed is a prerequisite for establishing joint attention, eye movements could afford a more direct and ecologically valid measurement. Second, eye movement parameters could grant an extra window on underlying processes. For instance, saccade latencies (i.e., the time from stimulus onset to the first saccade) can provide information about the amount of cognitive processing for each stimulus. Indeed, Hietanen (1999, 2002) suggested that head and eyes aligned or body and head aligned contain less information (than misaligned body parts) and therefore elicit no gaze cueing. In contrast, incongruent head and body orientations do elicit cueing. This would be the case because the multiple cues are processed in an allocentric frame of reference (i.e., related to the observed person). When eye and head or head and body are aligned, this would suggest that there is no implied attentional shift from the observed person and would thus not indicate a point of interest for the observer whereas misaligned eyes and head or head and body do indicate an implied attentional shift of the observed person. Therefore, we hypothesized that saccade latencies would be longer for aligned head and body than for maximally misaligned head and body.

### Methods

#### Participants

Seven students from the University of Leuven participated in the experiment (mean age of 22 years, two male students). All participants had normal or corrected-to-normal vision and were naïve with respect to the purpose of the study. Participation in this experiment was completely voluntary and prior to the experiment, every participant provided informed consent, conform to the ethical standards laid down in the 1964 Declaration of Helsinki.

#### Apparatus

A DELL Workstation PWS 370 Intel Pentium 4 3 GHz PC was used to display the stimuli on a 22-inch CRT color monitor with a refresh rate of 75 Hz, using the Experiment Builder v.1.10.1 software package (SR Research Osgood, ON, Canada). Participants were seated in a darkened room at a distance of 80 cm from the monitor. Head position was restrained using a chin-rest. Eye movements of both eyes were recorded using the Eyelink II video-based eye-tracker (SR Research Osgood, ON, Canada), at a rate of 250 Hz (using both pupil and corneal reflection). Recording was controlled by a second DELL Dimension 4700 Intel Pentium 4 3 GHz PC. Responses were registered via a computer mouse.

#### Stimuli

**FIGURE 1:**
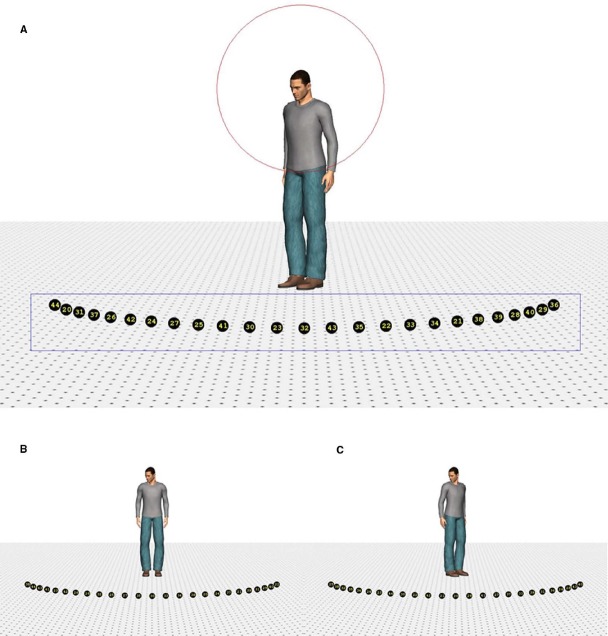
**Example of the stimuli. Stimulus with a head orientation of 40° to the left and a body orientation of 40° (A), 0° (B), and –40° (C).** The rectangle indicates the region of interest in which a fixation had to be recorded to switch to the response screen. The circle depicts the range outside which the landing position of a saccade had to fall to use its starting time to compute saccade latency.

Four different human characters (two male and two female) were used as stimuli. They were positioned on the ground surface of a scene, which contained a depth-perspective cue (Figure 1). The images of the characters subtended a visual angle of 9° in the vertical direction. The head of the characters subtended a visual angle of 1.1° × 0.7°, approximately. The characters were (individually) presented at the center of the screen. The ground surface contained 25 markers, black circles with a yellow number^1^ (subtending a visual angle of approximately 0.5°), positioned on a virtual circle surrounding the character. The markers were positioned on the circumference of the circle in such a way that the central marker was right in front of the character (i.e., a position of 0°). The rest of the markers were evenly divided on the circle (12 to the left and right of the middle marker) and separated from each other by 5° (on the circle in depth). Therefore, the total region of the circumference covered by the markers was 120° (60° on the left and on the right). Due to perspective, however, the actual on-screen separation between the markers decreased as they were further from the central marker.

Poser™ body modeling software (version 7.0, E-Frontier Inc.) was used to generate the four characters, two male and two female. Each of them wore different clothing and had a neutral face expression. To create smooth shading on the models, three “infinite” lights were used to illuminate the characters (one frontal light and two lights 30° to the left and right of the character positioned 45° above the viewer). Rendering of the images was done in color without shadows. The characters’ heads were tilted downward to ensure that they were facing the markers. By combining four head (40° left, 20° left, 20° right, 40° right) and five body orientations (40° left, 20° left, 0°, 20° right, 40° right), 20 different images were created for each character, yielding a total of 80 different stimuli. Eye and head orientation were always aligned.

#### Design

The experiment consisted of a 2 × 2 × 5 within-subjects design with spatial hemifield of the head (left or right), head orientation (20° or 40°) and body orientation (–40°, –20°, 0°, 20°, 40°) as within-subject factors, which were manipulated in a factorial way.

#### Procedure

**FIGURE 2:**
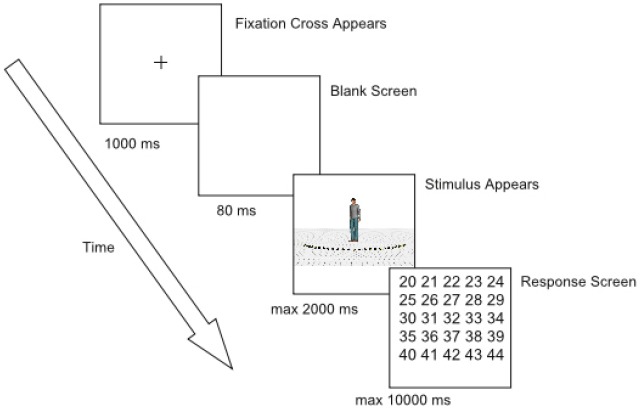
**Example of the trial sequence**.

Each trial began with the presentation of a fixation cross (subtending 0.72° × 0.72° of visual angle) for 1000 milliseconds (ms) at the same position as where the head of the character would be displayed (Figure 2). Then, the fixation cross disappeared and a blank screen was shown for 80 ms. After the blank screen, the character appeared together with the 25 markers and the ground surface. Participants were instructed to make an eye movement to the marker of which they perceived the character was looking at and to remember the number of this marker. Since eye movements were not yet recorded during practice trials, stimulus presentation lasted 600 ms. During the experimental session, stimulus presentation was gaze-contingent. If a fixation was detected within a predefined rectangular region of interest (subtending 22.6° × 3° of visual angle, Figure 1), an additional 400 ms was given to the participants to encode the number of the marker they were looking at. Subsequently, a grid with numbers 20–44 (subtending 6.9° × 7.3° of visual angle, approximately) was displayed and participants had to respond through a mouse click which number they thought the character was looking at. After responding, the next trial started. When no fixation was detected within 2000 ms from stimulus onset, an automatic switch to the response screen occurred. When no answer was given within 10000 ms after the onset of the response screen, the trial ended automatically (time-out response). Participants were encouraged to click outside the response grid when they did not encode the number of the marker or were not sure of their answer^2^ (“do not know” responses). No feedback was provided.

First, participants completed 20 practice trials to get acquainted with the task. These trials consisted of stimuli with a head orientation of 0° (i.e., looking straight forward) and all possible body orientations. Subsequently, they completed 10 blocks of 80 trials. At the beginning of each block, the pupil and corneal reflection were checked and the system was calibrated and validated according to the standard procedures (nine point calibration and validation). In each block, the 80 stimuli were presented in a random order. Taking a break was encouraged after every two blocks, but possible after every block. The experiment lasted approximately one and a half hour after which participants were debriefed.

### Results

Data preprocessing was done with custom code written in Python 2.6 and all subsequent analyses were carried out in R (R Core Team, 2014). All statistical analyses were performed in a Bayesian framework relying on Bayes factors (BFs) calculated using the BayesFactor package (Rouder and Morey, 2012; Rouder et al., 2012). BFs constitute a *relative* measure of evidence, quantifying how much more likely one statistical model is compared to another. For example, a BF of 3 for a statistical model including two main effects versus a statistical model including two main effects and their interaction indicates that the former model is three times more likely than the latter. This would indicate that no interaction is present in the data. It should be stressed that a BF does not constitute an absolute measure of model fit, but is *always* a *relative* measure of one model compared to another. For clarity, all BFs reported in this study are always relative to the *best fitting model* (i.e., the model that is most likely). Thus, a model for which the BF is 1 indicates the best fitting model (see Tables 1–4). BFs >1 then indicate how much more likely the best fitting model is compared to another model. All models for which BFs were computed were ANOVA-style models including random intercepts and slopes for participants. The models that were considered ranged from the simplest possible model (no main effect or interaction) to the most complex one (including all main effects and their interaction) and all that fell in between. BFs >3 are considered to be substantial evidence for the best fitting model over the other (Jeffreys, 1961). Please note that in all Tables all models for which BF >100 are collapsed under the term “all other models.”

**TABLE 1:** **Bayes factor analysis for the behavioral data**.

**Model**	**Bayes factor**
**40° Head orientation**	
Body orientation + hemifield	1
All other models	>100
**20° Head orientation**	
Body orientation + hemifield	1
Body orientation * hemifield	22
All other models	>100

Bayes factors are relative to the best fitting model (the model for which the Bayes factor is 1).*Indicates a model containing the main effects and the interaction between the variables.

#### Behavioral Data

Inspection of the raw data took place before applying any statistical test. “Do not know” and time-out responses were identified and removed from the raw data (1.8% of total). Subsequently, a criterion of a deviation of 30° or more from the correct response (i.e., six markers to the left or right from the correct response) was used to identify extreme or, rather, highly unlikely responses. These responses could be due to not remembering the number of the marker anymore or an inaccurate mouse click. This criterion yielded 19 data points to be extreme (0.3% of the data) and visual analysis of these points confirmed that these responses were probably due to a wrong transformation from coordinates to response location. Therefore, these data points were also removed from the data set.

For each head orientation (20° vs. 40°), a separate BF analysis was performed to assess which model fitted the data best^3^, summarized in Table 1. For both head orientations, the analysis indicated that models with main effects of body orientation and spatial hemifield were most likely.

**FIGURE 3:**
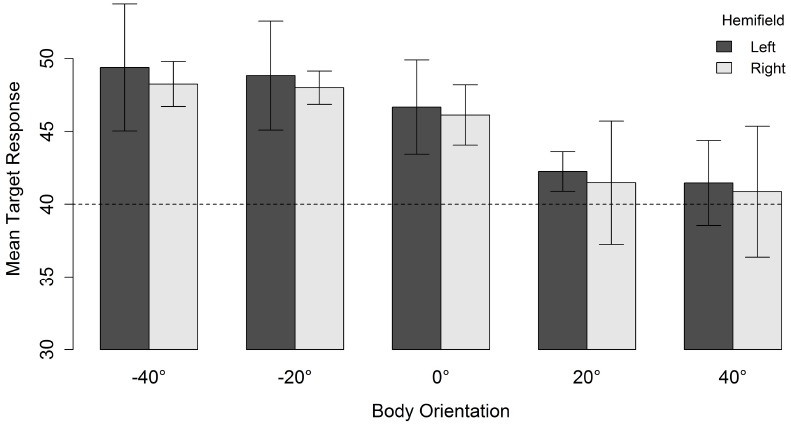
**Mean target response for a head orientation of 40° in function of body orientation and spatial hemifield.** Negative orientations indicate that the body is oriented in the direction opposite to the head. Error bars depict 95% confidence intervals according to the method of Cousineau (2005) adjusted with the correction suggested by Morey (2008). The dashed line indicates the veridical gazed-at target location of the character.

As can be seen in Figure 3, for a head orientation of 40°, participants perceived the character as looking further away from the veridical direction of the head (in the direction opposite to body orientation) as the misalignment between head and body orientation increased. When misalignment was maximal (i.e., head 40° and body –40°), the mean response location was 48.7°. Decreasing the misalignment between head and body until they both were aligned resulted in a mean response location of 41.1°. There was, therefore, a slight overall tendency to overestimate the character’s veridical gazed-at location (i.e., head and body congruent did not yield a mean response location of 40°). However, the perceived gaze direction of the characters was systematically biased in the direction opposite to the direction of the body relative to the head (i.e., an overshoot effect).

**FIGURE 4:**
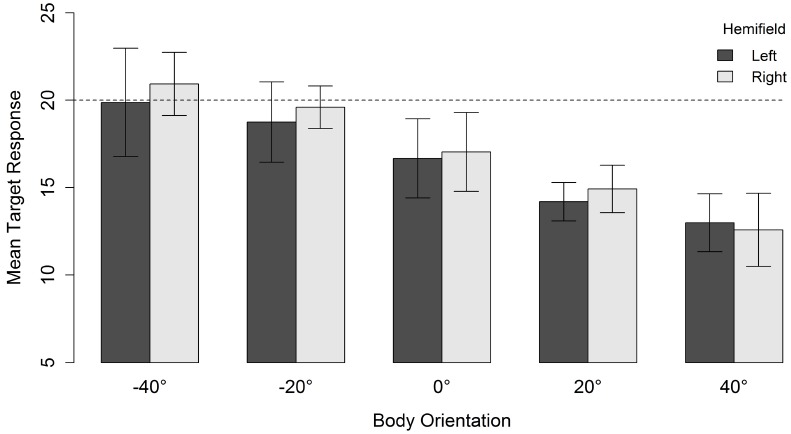
**Mean target response for a head orientation of 20° in function of body orientation and spatial hemifield.** Negative orientations indicate that the body is oriented in the direction opposite to the head. Error bars depict 95% confidence intervals according to the method of Cousineau (2005) adjusted with the correction of Morey (2008). The dashed line indicates the veridical gazed-at target location of the character.

For a head orientation of 20° there was, in contrast to a head orientation of 40°, a general tendency to underestimate the gaze direction of the character (Figure 4). When head and body were aligned (i.e., both 20°), the mean response location was 14.5°. Nevertheless, increasing the misalignment between body and head orientation yielded the same relative effect. The perceived gaze direction shifted in the direction opposite to the direction of the body relative to the head. This resulted in a mean response location of 20.4° when the body was oriented 40° in the direction opposite to the head. Orienting the body 40° in the same direction as the head also resulted in an estimated gazed-at location in the opposite direction of the body orientation and lower than the estimation of head and body aligned (i.e., an “overshoot” in a direction opposite to the “overshoot direction” observed for the other body orientations). Although there was an effect of spatial hemifield for both head orientations, note that this effect went in opposite directions for both head orientations and was very small compared to the effect of body orientation. Therefore, we do not consider it further.

Altogether, an overshoot effect was observed for both head orientations. Perceived gaze direction was systematically biased in the direction opposite to the body orientation. Furthermore, a head orientation of 20° was generally underestimated.

#### Eye movement Data

The eye movement data were analyzed in two ways. First, the angle of the first fixation within a predefined interest area relative to the fixation cross (centered on the character’s head) was computed and compared to the manual response. The goal of this analysis was to validate the manual responses. Indeed, to ensure we capitalized on the first estimate of perceived gaze direction, the eye movement data had to be in close agreement with the manual response. Second, saccade latencies were analyzed in function of head and body orientation and spatial hemifield. Specifically, the hypothesis that saccade latencies would be longer for aligned head and body orientations in comparison with maximally misaligned orientations was investigated.

***Validation of manual responses***

To validate manual responses, the angle between a fixation and the center of the character’s head was computed. This fixation was the first fixation of which the (Euclidian) distance to one of the targets was less than 1° of visual angle. The rationale behind this analysis was that if the angle between the first target fixated by the participants and the character’s head would show a high correspondence with the manual response, this would indicate that participants indeed reported the target number at which they looked. The correlation between this angle and the manual response^4^ was 0.92 (BF = 19). This suggests that there is a strong linear relationship between the looking angle and the subsequent response. However, this correlation does not inform us whether the manual response was equal to the looking angle. Therefore, the difference between the looking angle and the manual response was computed and a one sample *t*-test on this difference revealed that this difference was not credibly different from 0 (BF = 54 in favor of the null model).

In summary, a high agreement between the looking angle and the subsequent response of the participants was found. It is therefore unlikely that participants did not respond in accordance with where they looked.

***Saccade Latencies***

**FIGURE 5:**
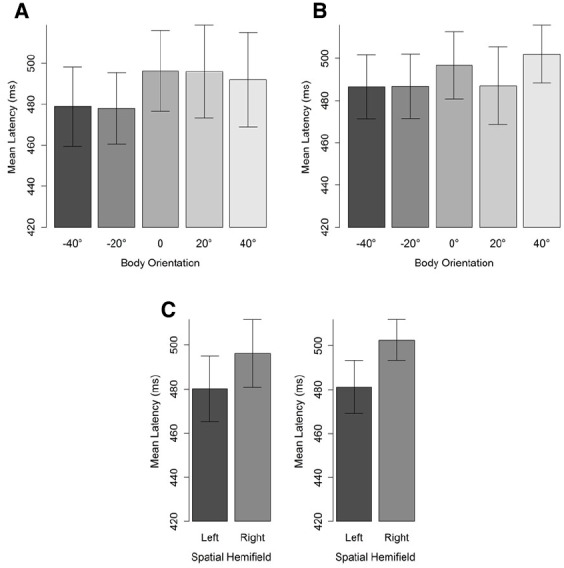
**Mean saccade latencies (ms) for a head orientation of 40° (A) and 20° (B) in function of body orientation.** Panel **(C)** depicts the hemifield effect. Negative body orientations denote orientations in the direction opposite to head orientation. Error bars depict 95% confidence intervals according to the method of Cousineau (2005) adjusted with the correction of Morey (2008).

The first saccade of which the landing position fell outside a region of 4° of visual angle (centered on the character’s head) was taken to compute saccade latencies (see Figure 1). Latencies were defined as the difference between starting time of the selected saccade and stimulus onset. Latencies lower than 100 ms were considered as anticipatory (i.e., the average latency between programming the eye movement and its onset is between 100 and 200 ms) and removed from the data set (two data points). A cut-off point at the upper tail of the distribution was set at 1000 ms, leading to the removal of 4% of the data. Mean saccade latencies were then subjected to a BF analysis, separately for 20° and 40° head orientation, with body orientation and hemifield as factors. For both head orientations, the best fitting models included only a main effect of spatial hemifield. That is, participants’ saccades were faster for the left than for the right hemifield. Critical for our predictions, however, no main effect of body orientation was observed (Table 2). Nevertheless, it should be noted that as the misalignment between body and head increased, saccade latencies decreased (Figure 5). Furthermore, simple comparisons between saccade latencies for aligned and maximally misaligned head and body orientations indicated that, for both head orientations, 0 always fell in the credible interval (95% posterior CI: [–12; 12] for a 20° head orientation and [–0.03; 25.58] for a 40° head orientation) yet for a head orientation of 40° the difference went in the predicted direction and the difference approached significance.

**TABLE 2:** **Bayes factor analysis for the saccade latencies**.

**Model**	**Bayes factor**
**40° Head orientation**	
Hemifield	1
Body orientation + hemifield	37
All other models	>100
**20° Head orientation**	
Hemifield	1
Body orientation + hemifield	5
All other models	>100

Bayes factors are relative to the best fitting model (the model for which the Bayes factor is 1).

### Discussion

The goal of Experiment 1 was to examine whether head and body orientation are integrated in a similar way as eye and head orientation in perceiving gaze direction. In analogy with the studies of Gibson and Pick (1963) and others, head and body orientation were independently manipulated and participants had to indicate where a computer-modeled character was looking. In line with the hypothesis, an overshoot effect was observed. The nature of this overshoot effect differed for the two head orientations. For a 40° head orientation, an *absolute* overshoot effect was observed. That is, when head and body were misaligned, people tended to judge the perceived gaze direction as being further away from the veridical gazed-at target location. Furthermore, aligned head and body orientations yielded a perceived gaze direction that mapped on to the character’s veridical gazed-at target location. In contrast, a 20° head orientation resulted in a *relative* overshoot effect. That is, when head and bodies were misaligned, perceived gaze direction again shifted in the direction opposite to body orientation. However, aligned head and body orientations yielded a perceived gaze direction that underestimated the veridical gazed-at target location. Only for maximally misaligned head and body orientations, perceived gaze direction was equal to the character’s veridical gazed-at target location. Why this discrepancy between the two head orientations was observed, is not clear. However, it has been observed before that observers underestimate veridical gaze direction for small head orientations (Masame, 1990; West et al., 2000; Kluttz et al., 2009).

As discussed in the introduction, a lot of research has been conducted regarding the critical features for perceiving gaze direction based on eye and head information. To date, the perception of body orientation has only been addressed to a limited extent. A recent adaptation study suggests that body orientation is represented by a multichannel system (Lawson et al., 2009). That is, distinct pools of cells would be coding for different body orientations, providing converging evidence with studies on macaque STS cells (Wachsmuth et al., 1994; Jellema and Perrett, 2003). Which body feature is critical for perceiving body orientation has not yet been studied. A plausible mechanism builds on the observations of Wilson et al. (2000). They showed that the perception of head orientation critically depends on the deviation of bilateral symmetry. Moreover, Lawson et al. (2011) recently documented that head orientation is represented similar to body orientation by a multichannel coding system. Thus, by extension, body orientation could be perceived in a similar way as head orientation, i.e., as the deviation from bilateral symmetry. Gaze direction would then be computed based on a combination of these two deviation cues. Nevertheless, this account does not explain why an overshoot effect was observed in Experiment 1. The literature on perceived gaze direction has not (yet) explicitly provided a theoretical account on the overshoot effect. However, the theoretical frameworks developed in the context of attentional orienting in response to gaze cues can be extrapolated to gaze estimation.

Two main theories have been proposed concerning how observers integrate information from the eyes, head, and body to estimate gaze direction and consequently allocate attention. Based on neurophysiological findings, Perrett et al. (1992) proposed a hierarchical model in which the attention of an observed person would be determined by the highest cue in the hierarchy only. For example, given eye, head, and body information only the eyes would be used as a cue. This implementation of a hierarchical model is contradicted not only by the findings of the present study but also by the seminal studies on the influence of head orientation on perceived gaze direction (Wollaston, 1824; Gibson and Pick, 1963; Anstis et al., 1969). In contrast with the model of Perrett et al. (1992), Langton (2000) and Langton et al. (2000) suggested that these cues are integrated in parallel having independent and additive effects on perceived direction of attention. This kind of model is able to predict an overshoot effect if body orientation is effectively taken into account into the additive combination of all available cues. Indeed, a recent study by Otsuka et al. (2014) showed perceived gaze direction can be modeled by a weighted sum of eye and head orientation information.

The model Hietanen (1999, 2002) proposes based on his own results provides an interesting framework. As has been discussed above, he argues that the different cues are not integrated in a frame of reference centered on the observer (egocentric) but in a frame centered on the observed at person (allocentric). This would imply that, when the orientation of the eyes and head are congruent (for any head orientation), this does not indicate a shift in attention of the looker (because he/she is looking straight ahead, but not necessarily in the direction of the observer) and, hence, elicits no cueing as Hietanen (1999) observed. Similarly, Hietanen (2002) reported no gaze cueing for congruent head and body orientations. Furthermore, incongruent eye and head or head and body orientations would imply a shift of attention. If computing another person’s attention happens in an allocentric frame of reference, then his/her gaze direction is presumably computed similarly. Since incongruent head and body orientations suggest a shift of the lookers’ attention, increased misalignment between head and body possibly generates a stronger directional spatial code than for the congruent head and body orientations. This stronger spatial code could then result in an overshoot effect as observed in Experiment 1. Putative evidence for this stronger spatial code could be the small differences between saccade latencies for congruent and incongruent head and body orientations observed in Experiment 1.

One possibility how allocentric coding may induce a stronger directional spatial code is by the activation of implied motion (Freyd, 1983; Verfaillie and d’ Ydewalle, 1991; Pomianowska et al., 2012). Indeed, an incongruent head and body orientation could indicate a rotating action from a resting posture. Implied motion may signal an intentional component of the looker which subsequently influences the perceived gaze direction of the observed person. For example, observers could implicitly assume that the eyes are oriented further than the head when there is a stronger intentional component present in the stimulus (i.e., when the body and head are gradually more and more misaligned). Experiment 2 was set out to test this hypothesis.

## Experiment 2

The goal of Experiment 2 was to examine the hypothesis that people implicitly assume that when the head and body are misaligned more and more, the eyes are oriented further than the head because of the stronger intentional component present in the stimulus (maybe due to the activation of implied motion). This could be a possible explanation of why an overshoot effect was observed in Experiment 1 (on top of a general bias in perceived gaze direction). To address this hypothesis, the stimulus was now presented from a side view (making eye information less salient) and head orientation of the character was manipulated in a vertical manner. If people indeed assume that gaze direction and head orientation are not equal, the hypothesis was that perceived gaze would be higher or lower than the character’s veridical gazed-at location, especially for large head orientations since these contain a stronger intentional component. More specifically, the rationale was that, if observers assume that the eyes are not aligned with the head as the intentional component in the stimulus rises, this would yield a more pronounced shift in perceived gaze for larger than for smaller head orientations. From a statistical viewpoint, this implies that the estimated slope for the relation between veridical head orientation and perceived gaze direction is reliably different from 1. If the intentional component does not affect perceived gaze direction, the prediction is that the slope does not differ from 1, yet the intercept can differ from 0.

### Methods

#### Participants

Nine students of the University of Leuven participated in the study (mean age 21, three male students). All participants had normal vision and were naïve with respect to the goal of the study. They all signed an informed consent prior to participation.

#### Apparatus

The equipment was the same as in Experiment 1.

#### Stimuli

**FIGURE 6:**
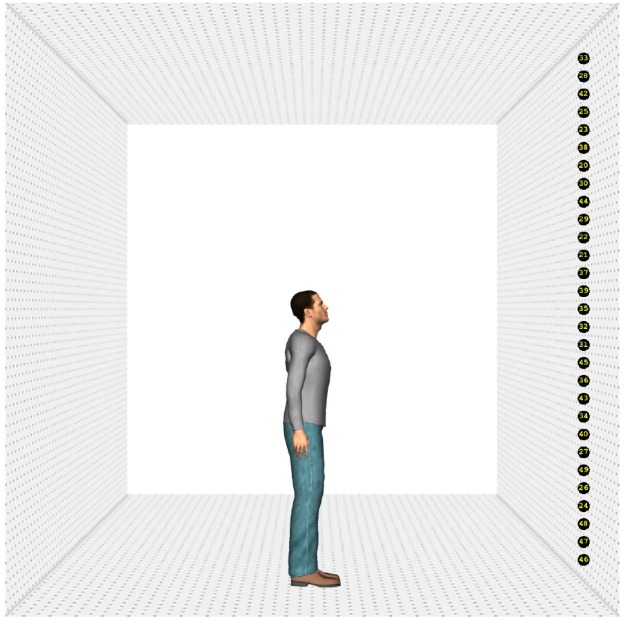
**An example of the stimuli of Experiment 2.** The character’s head is tilted 20° upward.

One of the four characters from Experiment 1 was picked and shown from a sagittal orientation (Figure 6). Thirty-one different stimuli were generated by increasing or decreasing the head orientation of the character by 2°, resulting in a range of head orientations from 30° downward to 30° upward. The ground surface was used to create a room-like setting by pasting it to the left, right and above the character.

Targets were added on the surface in front of the character’s head, i.e., on the right side of the display. The distance from the character to the targets was not the same as in Experiment 1 because this distance did not allow for using large angles in the upper and lower part of the display. The targets were equally spaced on the wall, 0.6° of visual angle apart and each subtended 0.4° of visual angle, ranging from 44° downward to 44° upward. In other words, targets reached from the ceiling to the floor.

#### Design

Head orientation was manipulated within-subjects and treated as a continuous variable.

#### Procedure

The procedure of Experiment 2 was the same as Experiment 1 except as noted here. The time for a trial to automatically switch to the response screen was reduced from 2000 to 1000 ms. Prior to the start of the experiment, participants completed a practice block of 31 trials in which every stimulus was presented once in a random order. The experimental sessions consisted of five blocks of 62 trials. Every stimulus was repeated twice in a random order. After every block, participants had the opportunity to take a small break. The system was recalibrated every block, even if participants did not wish to take a break.

### Results

#### Behavioral Data

The data were cleaned in the same way as in Experiment 1. About 4% of the data was removed due to “do not know” and time-out responses. Subsequently, a criterion of responses higher or lower than 10° if the character was looking downward or upward, respectively, was used to additionally clean the data (i.e., similar to the 30° deviation criterion in Experiment 1). This led to the removal of an additional 5% of the data. The data were analyzed with a Bayesian mixed-effects model including a random effect for participants (Rouder et al., 2012; see Rouder and Morey, 2012, for examples). Again, all models from the simplest to the most complex one were considered. Head orientation of the character was coded from –30° (downward) to 30° (upward) as was the perceived looking angle (–44° to 44°). The goal of this analysis was to examine (1) whether a character that was objectively looking straight ahead also was perceived as such (i.e., whether the intercept was different from 0 or not) and (2) whether an increase in head orientation resulted in a similar increase in perceived looking angle (i.e., whether the slope was different from one or not).

**FIGURE 7:**
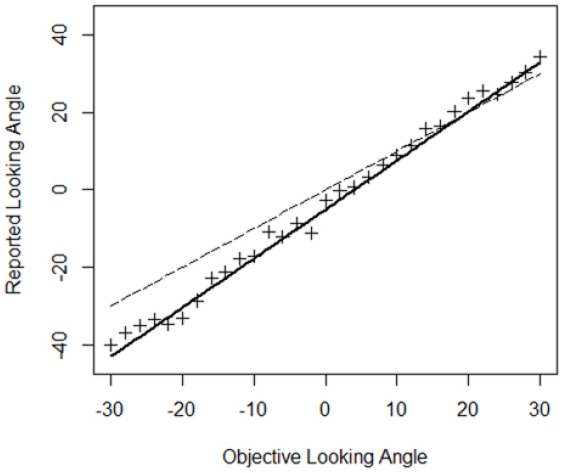
**Relationship between objective and reported looking angle.** Crosses are the data points. The dashed line indicates a “veridical” one-to-one relationship. The thick black line depicts the predictions according to the model in Table 4.

A model with objective looking angle as single predictor was fit to the data since this model was preferred in the BF analysis (Table 3). Table 4 depicts the parameter estimates of the fixed effects of this model^5^ and the associated 95% posterior credible intervals. The intercept is reliably different from 0 indicating that a computer-defined character looking straight ahead was not perceived as such, but looking a bit more downward. A character looking a bit upward was thus perceived as gazing straight ahead. Individual regressions showed that this was the case for seven out of nine participants. Furthermore, the slope associated with objective looking angle is reliably different from one as the 95% credible interval indicates. An increase of 1° in objective looking angle is associated with an expected increase in perceived looking angle of 1.27°. Thus, objective looking angles were overestimated and this overestimation was larger for larger head orientations. Note however that, due to the downward bias, in the upper hemifield the overestimation actually yields perceived looking angles on the “veridical” line for most of the tested looking angles. Figure 7 depicts the relation between objective looking angle and perceived looking angle.

**TABLE 3:** **Bayes Factor analysis for the behavioral data of Experiment 2**.

**Model**	**Bayes factor**
Angle	1
Angle + hemifield	16
All other models	>100

Bayes factors are relative to the best fitting model (the model for which the Bayes factor is 1).

**TABLE 4:** **Parameter estimates for a model including only Angle (coded from –30° to 30°)**.

**Predictor**	**Estimate**	95% posterior CI	
		Lower	Upper
Intercept	–3.72	–7.20	–0.21
Angle	1.27	1.25	1.29

In summary, these analyses indicate that (1) a computer-defined 0° looking angle is not perceived as such and (2) objective looking angles are consistently overestimated and this overestimation is larger for larger head orientations.

#### Eye movement Data

The behavioral responses were again validated with the same method as in Experiment 1 by taking the angle between the fixation and the character’s head as an indication where the participant was looking. The correlation between this angle and the manual response was again high (*r* = 0.89, BF = 52). The difference between the physical angle of the targets and the angle of the fixation again was not credibly different from 0 (BF = 40 in favor of the null model). Again, this indicates that participants’ responses were in accordance with the target at which they were looking.

### Discussion

The results of Experiment 2 indicate that there is a tendency to judge a computer-defined 0° looking angle as looking slightly downward. Furthermore, since the slope associated with objective looking angle was reliably different from one, veridical gaze direction was more strongly overestimated as the degree of intentionality in the stimulus rose (i.e., for large head orientations upward or downward). These results thus are in line with the predictions derived from the hypothesis that, when the stimulus has an implied intentional component, observers do not assume that gaze direction is equal to head orientation when they have to determine the gaze direction of a looker in the absence of horizontal information from the eyes.

## General Discussion

The goal of this paper was to study whether body information is integrated with head information to estimate the direction of another person’s gaze. In Experiment 1, body orientation was manipulated for two different head orientations. It was shown that body orientation indeed has an influence on perceived gaze direction and an overshoot effect was observed for both head orientations. That is, increasing the misalignment between head and body orientation yielded a perceived gaze direction in the direction opposite to the body orientation. In Experiment 2, a possible explanation for the observed overshoot effect was explored. Indeed, observers may assume that the looker does not have his/her eyes aligned with his head when there is a stronger intentional component present in the stimulus. In Experiment 2, the stimulus was presented from a side view and only head orientation was varied in a vertical direction. The results were in line with the predictions derived from the hypothesis that observers implicitly assume that gaze direction and head orientation are incongruent. Gaze direction was consistently overestimated in both the upper and lower hemifield. Importantly, the degree of overestimation was larger when the stimulus contained a stronger intentional component.

From a methodological point of view, the experiments have two implications. First, the eye movement results of both experiments indicate that it is valid to use this paradigm in future studies. Participant’s manual responses did not differ significantly from the target at which they were looking before their response. The advantage of using eye movements instead of manual responses is that it capitalizes on the first estimate of perceived gaze direction after stimulus onset. Indeed, measuring eye movements to the object gazed at by the looker is a more direct overt measure of joint attention (at least more direct than the manual responses used in most other paradigms).

Second, the results of Experiment 1 have some implications for further research on gaze perception in general. Indeed, since body orientation has an influence on perceived gaze direction, it is important to control for the orientation of the body in order to disentangle the possible influence of head/eye orientation and body orientation when the stimulus also includes the torso or full body. One example is the study of Poppe et al. (2007). The goal of this study was to examine the perception of head orientation in a triadic gaze task. The stimulus also included the torso, but head orientation was manipulated independently of body orientation. Therefore, their results are possibly confounded with the influence of body orientation on perceived gaze direction.

The main limitation of both experiments is that 2-D computer models were used as stimuli instead of 3-D live models. Indeed, differences have been reported between 2-D and 3-D stimuli (see Kluttz et al., 2009, for an overview), especially for turned heads. In the ideal case, a future study could repeat Experiment 1 with 3-D live models although this presents a lot more methodological challenges compared to the stimuli used here. Nevertheless, this would greatly contribute to the ecological validity of the present study.

It should be noted that one could argue that the results we obtained in Experiment 2 might not be specific to gaze stimul *per se*, but reflect a general overestimation effect in function of the increasing discrepancy between the head orientation and the body posture. Whereas a non-face control stimulus is often employed in the gaze cueing literature to assess the reflexive nature of the attentional shift (Frischen et al., 2007), this is almost never done in the gaze estimation literature. The degree to which this methodological concern impacts the current findings and those in the gaze estimation literature more generally has to be resolved in future studies. To our knowledge, there is one study, however, that sheds some light on this issue. Hudson et al. (2009) examined how perceived gaze direction impacts the judgment of how far a an agent’s head had rotated. In this study, a non-face control stimulus was also used. The results indicated that, whereas an effect of perceived gaze direction was found for the face stimuli, none was observed for the non-face control stimuli, implying that the observed effect was specific to gaze stimuli.

Due to the static nature of our stimuli, the overshoot effect reported in this study is necessarily limited to implied attentional shifts. The question remains whether the use of dynamic stimuli (e.g., containing motion-based attentional shifts) would affect the integration of eye/head and body information such that qualitatively different results would be observed. For example, dynamic stimuli might convey more information regarding the nature and type of attentional shift of the looker which could attenuate the overshoot effect as observed in this study for static stimuli. Indeed, Bock et al. (2008) report that observers are quite accurate in a triadic gaze task when they are allowed to follow the gaze of a live model.

The results of these experiments fit best with the model (Hietanen, 1999, 2002) proposed for how the different directional cues of eye, head, and body orientation are integrated. From a looker-related (allocentric) frame of reference, eye orientation would be processed relative to head orientation and head orientation relative to body orientation to determine whether the looker has an averted attention. This model suggests that the different cues are *always* integrated with each other in order to determine the looker’s attention. It is possible, however, that the extent to which these cues are integrated depends on the context. For example, if both eye and head information already indicate that the looker has averted his attention one can ask whether head orientation will still be referenced to body orientation. However, in the absence of eye information or when eye and head orientation are congruent, it would be informative to reference head to body orientation. The viability of this model could be addressed by conducting an experiment in which eye, head, and body orientation are manipulated independently. Seyama and Nagayama (2005) conducted such a study and their findings indicate that congruent eye–head relationships and incongruent eye–torso relationships both trigger attentional shifts. They never consider the head–torso relationship, however. A future study could manipulate eye, head, and body orientation independently to study whether there still is an influence of body orientation when eye and head orientation are not congruent.

## Conclusion

The results of this study indicate that body information is integrated with head information to perceive where another person is looking. In the first experiment, head and body orientation were independently manipulated and an overshoot effect was observed. This overshoot effect was explained in terms of an allocentric coding mechanism for computing another person’s gaze. This mechanism would activate a stronger directional spatial code as the stimulus has a stronger intentional component due to incongruent head and body orientations. This spatial code could be exerted by the implicit assumption that gaze direction and head orientation are not equal given an intentional component in the stimulus. This was confirmed in the second experiment. In the absence of horizontal eye information, overestimation of gaze direction was larger when the intentional component in the stimulus became stronger. These results have implications for theoretical frameworks that explain how different directional cues are integrated to estimate another person’s gaze.

### Conflict of Interest Statement

The authors declare that the research was conducted in the absence of any commercial or financial relationships that could be construed as a potential conflict of interest.

## Footnotes

1 The numbers ranging from 20 to 44 were picked. The only reason to do this was to achieve double digits everywhere and a more or less perceptually similar number in each circle. The order of numbers on the markers was randomized for every stimulus.

2 Additionally, participants were also asked to click in the middle of the number. This was done because the coordinates of the mouse clicks were the output data and these coordinates were transformed offline to the associated target number. If a region in between two numbers was clicked it would be possible that the response was transformed to a wrong number.

3 Because of the fact that, depending on the level of head orientation, the meaning of body orientation is different (e.g., 20° body orientation is congruent for 20° head orientation but not for 40° head orientation), it was decided to carry out two separate analyses.

4 Due to perspective, the angle at which the targets were placed did not always match the angle with which the target number was associated. For example, a target at 60° was not placed at a 60° angle from the character’s head. Because the angle computed from the eye movement data does not take this into account, the veridical angle at which targets were placed was used.

5 Because some participants were at ceiling for the lower hemifield, this analysis was done again without these data points. This revealed no differences from the analysis reported here. Furthermore, because of the ambiguity of the subjective point at which participants perceive the character as gazing straight ahead, data points with an objective looking angle around 0° were possibly not consistently judged as looking upward or downward. Therefore, the subjective point at which the avatar was judged to be gazing straight ahead was computed for every participant (based on individual regressions) and this range of looking angles was removed from the data of every participant. Again, this did not change the conclusions.

## References

[B1] Ando S. (2002). Luminance-induced shift in the apparent direction of gaze. Perception 31, 657–674. 10.1068/p3332 12092793

[B2] Ando S. (2004). Perception of gaze direction based on luminance ratio. Perception 33, 1173–1184. 10.1068/p5297 15693663

[B3] Anstis S. M. Mayhew J. W. Morley T. (1969). The perception of where a face or television “portrait” is looking. Am. J. Psychol. 82, 474–489. 10.2307/1420441 5398220

[B4] Argyle M. Cook M. (1976). Gaze and Mutual Gaze. Cambridge: Cambridge University Press.

[B5] Baron-Cohen S. (1994). How to build a baby that can read minds: cognitive mechanisms in mindreading. Curr. Psychol. Cogn. 13, 513–552.

[B6] Bayliss A. P. Bartlett J. Naughtin C. K. Kritikos A. (2011). A direct link between gaze perception and social attention. J. Exp. Psychol. Hum. Percept. Perform. 37, 634–644. 10.1037/a0020559 21038995

[B7] Bock S. W. Dicke P. Thier P. (2008). How precise is gaze following in humans? Vision Res. 48, 946–957. 10.1016/j.visres.2008.01.011 18294671

[B8] Carlin J. D. Calder A. J. Kriegeskorte N. Nili H. Rowe J. B. (2011). A head view-invariant representation of gaze direction in anterior superior temporal sulcus. Curr. Biol. 21, 1817–1821. 10.1016/j.cub.2011.09.025 22036180 PMC3267037

[B9] Cline M. G. (1967). The perception of where a person is looking. Am. J. Psychol. 80, 41–50. 10.2307/1420539 6036357

[B10] Cousineau D. (2005). Confidence intervals in within-subject designs: a simpler solution to Loftus and Masson’s method. Tutor. Quant. Methods Psychol. 1, 42–45.

[B11] Driver J. Davis G. Ricciardelli P. Kidd P. Maxwell E. Baron-Cohen S. (1999). Gaze perception triggers reflexive visuospatial orienting. Vis. Cogn. 6, 509–540. 10.1080/135062899394920

[B12] Duchaine B. Jenkins R. Germine L. Calder A. J. (2009). Normal gaze discrimination and adaptation in seven prosopagnosics. Neuropsychologia 47, 2029–2036. 10.1016/j.neuropsychologia.2009.03.011 19467353

[B13] Emery N. J. (2000). The eyes have it: the neuroethology, function and evolution of social gaze. Neurosci. Biobehav. Rev. 24, 581–604. 10.1016/S0149-7634(00)00025-7 10940436

[B14] Freyd J. J. (1983). The mental representation of movement when static stimuli are viewed. Percept. Psychophys. 33, 575–581. 10.3758/BF03202940 6622194

[B15] Friesen C. Kingstone A. (1998). The eyes have it! Reflexive orienting is triggered by nonpredictive gaze. Psychon. Bull. Rev. 5, 490–495. 10.3758/BF03208827

[B16] Frischen A. Bayliss A. P. Tipper S. P. (2007). Gaze cueing of attention. Psychol. Bull. 133, 694–724. 10.1037/0033-2909.133.4.694 17592962 PMC1950440

[B17] George N. Driver J. Dolan R. J. (2001). Seen gaze-direction modulates fusiform activity and its coupling with other brain areas during face processing. Neuroimage 13, 1102–1112. 10.1006/nimg.2001.0769 11352615

[B18] Gibson J. J. Pick A. D. (1963). Perception of another person’s looking behavior. Am. J. Psychol. 76, 386–394. 10.2307/1419779 13947729

[B19] Hanna J. E. Brennan S. E. (2007). Speakers’ eye gaze disambiguates referring expressions early during face-to-face conversation. J. Mem. Lang. 57, 596–615. 10.1016/j.jml.2007.01.008

[B20] Hietanen J. K. (1999). Does your gaze direction and head orientation shift my visual attention? Neuroreport 10, 3443–3447. 10.1097/00001756-199911080-00033 10599859

[B21] Hietanen J. K. (2002). Social attention orienting integrates visual information from head and body orientation. Psychol. Res. 66, 174–179. 10.1007/s00426-002-0091-8 12192446

[B22] Hudson M. Liu C. H. Jellema T. (2009). Anticipating intentional actions: the effect of eye gaze direction on the judgment of head rotation. Cognition 112, 423–434. 10.1016/j.cognition.2009.06.011 19615675

[B23] Jeffreys H. (1961). Theory of Probability. Oxford: Oxford University Press.

[B24] Jellema T. Perrett D. I. (2003). Perceptual history influences neural responses to face and body postures. J. Cogn. Neurosci. 15, 961–971. 10.1162/089892903770007353 14614807

[B25] Kluttz N. L. Mayes B. R. West R. W. Kerby D. S. (2009). The effect of head turn on the perception of gaze. Vision Res. 49, 1979–1993. 10.1016/j.visres.2009.05.013 19467254

[B26] Langton S. R. (2000). The mutual influence of gaze and head orientation in the analysis of social attention direction. Q. J. Exp. Psychol. A Hum. Exp. Psychol. 53, 825–845. 10.1080/713755908 10994231

[B27] Langton S. R. H. Honeyman H. Tessler E. (2004). The influence of head contour and nose angle on the perception of eye-gaze direction. Percept. Psychophys. 66, 752–771. 10.3758/BF03194970 15495901

[B28] Langton S. R. H. Watt R. J. Bruce V. (2000). Do the eyes have it? Cues to the direction of social attention. Trends Cogn. Sci. 4, 50–59. 10.1016/S1364-6613(99)01436-9 10652522

[B29] Lawson R. P. Clifford C. W. G. Calder A. J. (2009). About turn: the visual representation of human body orientation revealed by adaptation. Psychol. Sci. 20, 363–371. 10.1111/j.1467-9280.2009.02301.x 19254238

[B30] Lawson R. P. Clifford C. W. G. Calder A. J. (2011). A real head turner: horizontal and vertical head directions are multichannel coded. J. Vis. 11, 17. 10.1167/11.9.17 21873615

[B31] Manera V. Schouten B. Becchio C. Bara B. G. Verfaillie K. (2010). Inferring intentions from biological motion: a stimulus set of point-light communicative interactions. Behav. Res. Methods 42, 168–178. 10.3758/BRM.42.1.168 20160297

[B32] Maruyama K. Endo M. (1983). The effect of face orientation upon apparent direction of gaze. Tohoku Psychol. Folia 42, 126–138.

[B33] Masame K. (1990). Perception of where a person is looking: overestimation and underestimation of gaze direction. Tohoku Psychol. Folia 49, 33–41.

[B34] Morey R. (2008). Confidence intervals from normalized data: a correction to Cousineau (2005). Tutor. Quant. Methods Psychol. 4, 61–64.

[B35] Noll A. M. (1976). The effects of visible eye and head turn on the perception of being looked at. Am. J. Psychol. 89, 631–644. 10.2307/1421462 1020765

[B36] Otsuka Y. Mareschal I. Calder A. J. Clifford C. W. G. (2014). Dual-route model of the effect of head orientation on perceived gaze direction. J. Exp. Psychol. Hum. Percept. Perform. 40, 1425–1439. 10.1037/a0036151 24730742 PMC4120707

[B37] Perrett D. I. Hietanen J. K. Oram M. W. Benson P. J. (1992). Organization and functions of cells responsive to faces in the temporal cortex. Philos. Trans. R. Soc. Lond. B Biol. Sci. 335, 23–30. 10.1098/rstb.1992.0003 1348133

[B38] Pomianowska I. Germeys F. Verfaillie K. Newell F. N. (2012). The role of social cues in the deployment of spatial attention: head–body relationships automatically activate directional spatial codes in a Simon task. Front. Integr. Neurosci. 6:4. 10.3389/fnint.2012.00004 22347172 PMC3269793

[B39] Pönkänen L. M. Hietanen J. K. (2012). Eye contact with neutral and smiling faces: effects on autonomic responses and frontal EEG asymmetry. Front. Hum. Neurosci. 6:122. 10.3389/fnhum.2012.00122 22586387 PMC3343319

[B40] Poppe R. Rienks R. Heylen D. (2007). Accuracy of head orientation perception in triadic situations: experiment in a virtual environment. Perception 36, 971–979. 10.1068/p5753 17844963

[B41] R Core Team. (2014). R: A Language and Environment for Statistical Computing. R Foundation for Statistical Computing, Vienna, Austria Available at: http://www.R-project.org/

[B42] Ricciardelli P. Baylis G. Driver J. (2000). The positive and negative of human expertise in gaze perception. Cognition 77, B1–B14. 10.1016/S0010-0277(00)00092-5 10980254

[B43] Rouder J. N. Morey R. D. (2012). Default Bayes factors for model selection in regression. Multivariate Behav. Res. 47, 877–903. 10.1080/00273171.2012.734737 26735007

[B44] Rouder J. N. Morey R. D. Speckman P. L. Province J. M. (2012). Default Bayes factors for ANOVA designs. J. Math. Psychol. 56, 356–374. 10.1016/j.jmp.2012.08.001

[B45] Seyama J. Nagayama R. (2005). The effect of torso direction on the judgement of eye direction. Vis. Cogn. 12, 103 10.1080/13506280444000111

[B46] Sinha P. (2000). Here’s looking at you, kid. Perception 29, 1005–1008. 10.1068/p2908no 11145079

[B47] Staudte M. Crocker M. W. (2011). Investigating joint attention mechanisms through spoken human–robot interaction. Cognition 120, 268–291. 10.1016/j.cognition.2011.05.005 21665198

[B48] Staudte M. Crocker M. W. Heloir A. Kipp M. (2014). The influence of speaker gaze on listener comprehension: contrasting visual versus intentional accounts. Cognition 133, 317–328. 10.1016/j.cognition.2014.06.003 25079951

[B49] Symons L. A. Lee K. Cedrone C. C. Nishimura M. (2004). What are you looking at? Acuity for triadic eye gaze. J. Gen. Psychol. 131, 451–469. 15523825 PMC2564292

[B50] Todorović D. (2006). Geometrical basis of perception of gaze direction. Vision Res. 46, 3549–3562. 10.1016/j.visres.2006.04.011 16904157

[B51] Todorović D. (2009). The effect of face eccentricity on the perception of gaze direction. Perception 38, 109–132. 10.1068/p5930 19323141

[B52] Verfaillie K. d’ Ydewalle G. (1991). Representational momentum and event course anticipation in the perception of implied periodical motions. J. Exp. Psychol. Learn. Mem. Cogn. 17, 302–313. 10.1037/0278-7393.17.2.302 1827832

[B53] Wachsmuth E. Oram M. W. Perrett D. I. (1994). Recognition of objects and their component parts: responses of single units in the temporal cortex of the macaque. Cereb. Cortex 4, 509–522. 10.1093/cercor/4.5.509 7833652

[B54] Watt R. Craven B. Quinn S. (2007). A role for eyebrows in regulating the visibility of eye gaze direction. Q. J. Exp. Psychol. 60, 1169–1177. 10.1080/17470210701396798 17676550

[B55] West R. W. (2010). Differences in the perception of monocular and binocular gaze. Optom. Vis. Sci. 87, E112–E119. 10.1097/OPX.0b013e3181ca345b 20035243

[B56] West R. W. (2011). Perceived direction of gaze from eyes with dark vs. light irises. Optom. Vis. Sci. 88, 303–311. 10.1097/OPX.0b013e3182059ef3 21150679

[B57] West R. W. Salmon T. O. Sawyer J. K. (2000). Influence of the epicanthal fold on the perceived direction of gaze. Optom. Vis. Sci. 85, 1064–1073. 10.1097/OPX.0b013e31818b963b 18981921

[B58] Wilson H. R. Wilkinson F. Lin L. M. Castillo M. (2000). Perception of head orientation. Vision Res. 40, 459–472. 10.1016/S0042-6989(99)00195-9 10820605

[B59] Wollaston W. H. (1824). On the apparent direction of eyes in a portrait. Philos. Trans. R. Soc. Lond. 114, 247–256. 10.1098/rstl.1824.0016

